# Supplementary material for: Predicting the spatial pattern of land use change and carbon storage in Xinjiang: A Markov-FLUS-InVEST model approach
Source: PLoS One. 2025 Apr 17;20(4):e0321929. doi: 10.1371/journal.pone.0321929 (PMC12005497; doi:10.1371/journal.pone.0321929)
Supplement: Attachment — Submitted filename: Response to Reviewers.docx [file pone.0321929.s002.docx]

I would like to express my heartfelt respect and gratitude to the expert for your meticulous and professional revision in your busy schedule, which makes the article more rigorous and further improved. Thanks to the editor and experts for the opportunity to revise. If there is any problem, please feel free to contact me at any time. I am very willing to make positive changes.

**Reviewer #1**

1. How the LULC maps for 2000, 2005, 2010, 2015, 2020 were generated? Using which classification method and satellite data? The accuracies during calibration and validation phase with confusion matrix should be presented.

**Response 1:** LULC maps come from the Resource and Environmental Science Data Platform. This data is based on remote sensing satellite images, Landsat 8 OLI, GF-2 and other remote sensing satellite data, and the land use data set is obtained through the construction of high-resolution remote sensing - unmanned aerial vehicle - ground survey observation technology system, combined with the human-computer interactive interpretation method based on geoscience knowledge. The classification accuracy and total accuracy were evaluated by confusion matrix. The comprehensive evaluation accuracy of the first type of land use is more than 93%, and the comprehensive accuracy of the second type classification is more than 90%, which meets the user's drawing accuracy of 1: 100,000 scale. We have combined the opinions of expert to provide a detailed explanation of the data sources in the article.

[1] Kuang W, Zhang S, Du G, et al. Monitoring Periodically National Land Use Changes and Analyzing Their Spatiotemporal Patterns in China During 2015-2020. Journal of Geographical Sciences. 2022; 32(9), 1705-1723.

[2] Liu J, Kuang W, Zhang Z, et al. Spatiotemporal Characteristics, Patterns, and Caus-es of Land-use Changes in China Since the Late 1980s Journal of Geographical Sciences. 2014; 24(2): 195-210.

2. What are the input parameters which were modified to generate future land use? What are the sources of those future input parameters? Details required.

**Response 2:** Input parameters include DEM, slope, aspect, rainfall, land surface temperature, net primary productivity of vegetation, luminous index, soil type, and geomorphology. These Data are from the Resource and Environmental Science Data Platform. We have made further supplementary explanations on the data sources in the manuscript in combination with the opinions of expert. Please see "2.2 Data" and "3.1 Multi-scenario land-use change simulation based on the FLUS model" for details.

3. What about the neighborhood factors for Natural growth scenario and ecological protection scenario?

**Response 3:** Neighborhood factors represented the difficulty of converting a land-use type into other land-use types, with a parameter range of 0-1. A score close to 1 indicates that the land-use type has a strong expansion ability. The natural growth scenario does not involve any intervention, so the neighborhood factor is set to 1. The ecological protection scenario is to strengthen the protection of forest land, grassland, water and other ecological land, while weakening the expansion capacity of other land types. Therefore, set the other land type parameter to 0.1. At the same time, we have made further additions to the presentation based on expert comments. Please see section 3.1 for details.

4. Which software were used for future land use and driving force analysis? Please mention that clearly with library or package name.

**Response 4:** We used GeoSOS-FLUS V2.4 Software for future land use simulation and Geodetector Software (beta) for ArcGlS Pro software for driving force analysis. We have taken the expert's advice in full and supplemented the package names in this article.

5. What do you mean by “geographic detector”? Is it a model? If yes, what is the name of the model? What are the inputs and output of the model?

**Response 5:** Geographic detector is a model called Geodetector. The model's input comprises the carbon stock value in Xinjiang, serving as the dependent variable (Y), alongside the values of ten distinct independent variable drivers (X1-X10). The output yields a contribution index for each driver, thereby quantifying the respective impact of these variables on the carbon stock. We have supplemented Geodetector with expert recommendations. For detailed modifications, refer to Section 3.3: Driving force analysis of spatiotemporal evolution of carbon storage based on Geodetector.

6. Abstract：

Expand “FLUS-InVEST”. Do not use abbreviations in abstract.

L No. 28: Driving factors include which parameters? Please specify.

**Response 6:** We have fully adopted the expert opinion and changed the abbreviation to the full name in the abstract. Furthermore, the driving factors were changed to specific parameters (land-use types). Please see the abstract section for details.

7. Introduction：

L No. 77-84: Delete “However, most existing studies have…factors on the spatiotemporal evolution characteristics of carbon storage.” As it is a repetition of L No. 69-76.

L No. 90-93: Delete “The results of this study provide scientific… and ecological decision-making.”

**Response 7:** We fully adopted the opinions of experts and deleted this part of the content.

8. Figure 1: Replace ‘KM’ with ‘km’ in the scalebar.

**Response 8:** We have replaced “KM” in the scale bar with “km” as recommended by expert.

9. Table 1 Write the source of DEM, rain, temperature, LST, NPP, nighttime light index, soil type, landform type with URL link.

**Response 9:** We have fully adopted the expert opinion and added source links for all the above data in Table 1.

10. L No. 125: Which interpolation method was used?

**Response 10:** Our resampling method used cubic convolution interpolation. We have provided additional explanations in the text. Detailed modification details can be found in "2.2. Data".

11. Replace “water areas” to “water bodies” throughout the manuscript.

**Response 11:** We fully adopted the expert opinion and replaced “water areas ” with “water bodies” throughout the manuscript.

12. Fig. 2 What do you mean by agrotype? Please maintain uniformity of the factor names throughout the manuscript. In table 1 - nighttime light index is written as Luminous index and landform type is written as geomorphology. Table 2, 3, 4; Figure 3 and 5: “Arable land” to “Cropland”, “Woodland” to “Forest land”

**Response 12:** The agrotype in the figure represents the soil types. We have redrawn the diagram and modified it to soil types. In addition, we have uniformly modified the full-text factor names according to expert recommendations.

13. Please expand the figure caption of figure 3 and 4 to make it self-explanatory.

**Response 13:** We have fully adopted the expert's advice and changed the title of the figure to make it more detailed and self-explanatory.

14. L No. 279: What do you mean by “high-value carbon storage areas”?

**Response 14:** “High-value carbon storage areas” means areas with high carbon storage. For better expression, we changed it to “high carbon storage areas”.

15. Fig. 6: Please enlarge the figures to make it legible.

**Response 15:** We have adopted expert advice and have enlarged the text in the figure.

**Reviewer #2**

The structure of this paper is clear and the methods used are flexible. It begins by analyzing the changes in land use in Xinjiang from 2000 to 2020, then simulates and predicts the land use in Xinjiang in 2035 under two scenarios using the FLUS-InVEST Model, and also analyzes the spatial and temporal changes in carbon storage in Xinjiang. Finally, it uses geographic model detectors to analyze the impact of driving factors. But it is recommended that the content be revised prior to publication so as to improve the quality of the article and its accessibility to readers. The main issues are as follows:

1. Are there any incorrect statements in the summary of results? Based on the research findings, the driving factors had the greatest carbon storage explanatory power (q=0.80), "The geographical detector analysis results showed that driving factors had the greatest carbon storage explanatory power (q=0.80)".

**Response 1:** Thanks for the expert advice. We have revised the statements in the summary of the results. “The geographical detector analysis results showed that land-use types had the greatest carbon storage explanatory power (q=0.80).”

2. In the Introduction, can you provide some models in the "among the many evaluation models" section to support your argument?

**Response 2:** We have fully followed the expert's advice and supplemented the introduction. Among the many models, including ARIES, GUMBO, MIMES, CITYgreen [1].

[1] Ma L, Jin T, Wen Y, Wu X, Liu G. The Research Progress of InVEST Model. Ecological Economy. 2015; 31(10), 126-131+179.

3. The sections from Introduction 69-76 and 77-84 are repetitive in meaning. Please modify this part and delete the redundant sentences. Also, please appropriately cite relevant literature to support your viewpoint.

**Response 3:** We have fully adopted the expert's advice, deleted the repetitive sentences in the introduction, and quoted some literature to support our viewpoint.

[1] Liang Y, Hu H, Crowther TW, Jörgensen RG, Liang C, Chen J, et al. Global decline in microbial-derived carbon stocks with climate warming and its future projections. National Science Review. 2024; 11: nwae330.

[2] Zhu K, He J, Tian X, Hou P, Wu L, Guan D, et al. Analysis of Evolving Carbon Stock Trends and Influencing Factors in Chongqing under Future Scenarios. Land. 2024; 13: 421.

4. The scale of the maps in Figure 1. Summary map of the study area and Figure 5. Local details of carbon storage should be "km". Please change it.

**Response 4:** Based on expert opinions, we have changed "KM" in all figures to "km".

5. The DEM legend in Figure 1. Summary map of the study area and Figure 5. Local details of carbon storage does not have units. Is it necessary to specify units for greater precision?

**Response 5:** We have fully adopted the expert opinions and redrawn the figures as required.

6. Please explain the principle behind the 0 and 1 in Table 2 for different scenario settings.

**Response 6:** We have fully adopted the expert's advice and made further supplementary explanations on the principle behind it. The transfer matrix is represented by 0 and 1. When conversion of one land use type to another is allowed, the corresponding value of the matrix is set to 1 and 0 when it is not allowed. The natural growth scenario does not involve any intervention, so the value is set to 1. The ecological protection scenario aims to strengthen the protection of ecological land such as forests, grasslands, and water bodies, so it does not allow them to change to other land use types, so it is set to 0. Please see section 3.1 for details.

7. In section 3.1, please explain the difference between "temperature" and "land surface temperature (LST)" in the selected driving factors.

**Response 7:** We have fully adopted the expert's advice. The difference between "temperature" and "Surface temperature (LST)" was added to section 3.1.

Temperature denotes the air's thermal state, specifically the air temperature recorded in a sheltered environment, which indicates the level of atmospheric warmth.

Land surface temperature pertains to the ground's thermal condition. It is measured at the boundary between the Earth's surface and the air and is influenced by various factors, including the type of terrain, vegetation, and soil moisture.

8. How were the driving factors chosen when using the Markov model in this study, and how were the driving factors chosen when building the geographical model detector?

**Response 8:** We are grateful to the expert for invaluable comments, which have charted the course for our future research endeavors. Following the expert' recommendations, we have implemented additional revisions within the domains of data, methodology, and discussion. The choice of driving factors when utilizing the Markov model was primarily informed by established research. Through rigorous experimentation, our study achieved a high degree of simulation precision (Kappa = 0.94), satisfying the stringent research criteria. Moreover, with the aim of enhancing the model's predictive accuracy and offering a robust framework for future factor selection, we employed the geographical detector to meticulously quantify the individual impact of the above driving factors on carbon stock levels. Given the intricate nature of carbon stock assessment, our future investigations will encompass a broader spectrum of considerations, including legislative and policy-oriented aspects.

9. Reference 20 has an incorrect citation format.

**Response 9:** We have modified the format of the 20th reference based on expert advice and have checked other references.

10. Why is there only a string diagram for land use transfer in Figure 3 for the natural growth scenario, but not for the ecological protection scenario?

**Response 10:** According to the opinions of expert, we have redrawn Figure 3 and added the string diagram of land use transfer under the ecological protection scenario. At the same time, we also modified Figure 4 to add the carbon storage change chart under the ecological protection scenario. Please see Figures 3 and 4 for detailed changes.

11. In "Xinjiang plays a vital role in global carbon storage because of its complex geological structure and mountain chains that include the Altai Mountains, Kunlun Mountains, and Tianshan Mountains", is it a bit stiff and disconnected? Suggest enriching the theoretical framework before introducing the topic.

**Response 11:** According to the suggestions of expert, we have deleted this part of the content and modified and improved the introduction of the study area. In addition, we also describe the theoretical framework in more detail before introducing the topic. Details can be found in 2.1 Overview of the study area.

Thanks again for the expert's contribution to this manuscript.

**Journal Requirements:**

1. When submitting your revision, we need you to address these additional requirements.

Please ensure that your manuscript meets PLOS ONE's style requirements, including those for file naming.

**Response 1:** We have checked and revised as required.

2. Thank you for stating in your Funding Statement:

Mengting Jin was supported by the Science and Technology Innovation Foundation of the Command Center for Comprehensive Survey of Natural Resources (KC20230015) and the China Geological Survey Project (DD20220962). She played a role in data collection, methodology, and project administration. Quan Xu was supported by the China Geological Survey Project (DD20240740). He played a role in data collection and methodology.

Please provide an amended statement that declares *all* the funding or sources of support (whether external or internal to your organization) received during this study, as detailed online in our guide for authors at http://journals.plos.org/plosone/s/submit-

now. Please also include the statement “There was no additional external funding received for this study.” in your updated Funding Statement.

Please include your amended Funding Statement within your cover letter. We will change the online submission form on your behalf.

**Response 2:** There has been no change in funding for this study. We have included a funding statement with the cover letter as requested.

3. We note that Figures 1, 2, 3, 4 and 5 in your submission contain map images which may be copyrighted. All PLOS content is published under the Creative Commons Attribution License (CC BY 4.0), which means that the manuscript, images, and Supporting Information files will be freely available online, and any third party is permitted to access, download, copy, distribute, and use these materials in any way, even commercially, with proper attribution. For these reasons, we cannot publish previously copyrighted maps or satellite images created using proprietary data, such as Google software (Google Maps, Street View, and Earth). For more information, see our copyright guidelines:http://journals.plos.org/plosone/s/licenses-and-copyright. We require you to either present written permission from the copyright holder to publish these figures specifically under the CC BY 4.0 license, or remove the figures from your submission:

a. You may seek permission from the original copyright holder of Figures1, 2, 3, 4 and 5 to publish the content specifically under the CC BY 4.0 license. We recommend that you contact the original copyright holder with the Content Permission Form (http://journals.plos.org/plosone/s/file?id=7c09/content-permission-form.pdf) and the following text:“I request permission for the open-access journal PLOS ONE to publish XXX under the Creative Commons Attribution License (CCAL) CC BY 4.0 (http://creativecommons.org/licenses/by/4.0/). Pleasebeaware that this license allows unrestricted use and distribution, even commercially, by third parties. Please reply and provide explicit written permission to publish XXX under a CC BY license and complete the attached form.” Pleaseupload the completed Content Permission Form or other proof of granted permissions as an "Other" file with your submission. In the figure caption of the copyrighted figure, please include the following text: “Reprinted from [ref] undera CC BY license, with permission from [name of publisher], original copyright[original copyright year].”

b. If you are unable to obtain permission from the original copyright holder to publish these figures under the CC BY 4.0 license or if the copyright holder’s requirements are incompatible with the CC BY 4.0 license, please either

i) remove the figure or ii) supply a replacement figure that complies with the CC BY 4.0 license. Please check copyright information on all replacement figures and update the figure caption with source information. If applicable, pleasespecify in the figure caption text when a figure is similar but not identical to the original image and is therefore for illustrative purposes only.

**Response 3:** All of our figures are created by us, utilizing public data that is properly cited and explained in the data source. Consequently, there are no copyright issues involved.

Thanks again to the experts and editor. If you have any questions, please feel free to contact us. We will respond positively.
