# Supplementary material for: Predicting the spatial pattern of land use change and carbon storage in Xinjiang: A Markov-FLUS-InVEST model approach
Source: PLoS One. 2025 Apr 17;20(4):e0321929. doi: 10.1371/journal.pone.0321929 (PMC12005497; doi:10.1371/journal.pone.0321929)
Supplement: Attachment — Submitted filename: Response_to_Reviewers_auresp_2.docx [file pone.0321929.s003.docx]

I would like to express my heartfelt respect and gratitude to the expert for your meticulous and professional revision in your busy schedule, which makes the article more rigorous and further improved. Thanks to the editor and experts for the opportunity to revise. If there is any problem, please feel free to contact me at any time. I am very willing to make positive changes.

**Journal Requirements:**

Please review your reference list to ensure that it is complete and correct. If you have cited papers that have been retracted, please include the rationale for doing so in the manuscript text, or remove these references and replace them with relevant current references. Any changes to the reference list should be mentioned in the rebuttal letter that accompanies your revised manuscript. If you need to cite a retracted article, indicate the article’s retracted status in the References list and also include a citation and full reference for the retraction notice.

**Response 1:** We would like to express our sincere appreciation to the editor for providing us with the opportunity to revise our manuscript. We have conducted a thorough review of all references and implemented the necessary revisions in compliance with the specified guidelines. Upon further investigation, it was noted that references 9 and 10 are not indexed in the Web of Science database; however, they are available through the CNKI (China National Knowledge Infrastructure). As a result, we have replaced these references with suitable alternatives, ensuring that the original text's integrity and content remain intact.

**Reviewer #1**

1. The authors have responded to all my questions satisfactorily. The revised version may be accepted for publication.

**Response 1:** We are sincerely grateful to the esteemed reviewer for granting us this invaluable opportunity, which we consider both a privilege and a profound responsibility.

**Reviewer #2**

1. The author has earnestly considered and meticulously revised the manuscript in response to the reviewers' comments. After examination, I am fundamentally satisfied with the revisions made by the author in the article. Through the revisions, the quality of the article has also enhanced. Nevertheless, it is proposed that the author could appropriately depict the characteristics of spatial distribution and incorporate regional variations in the analysis, rather than merely focusing on the description of land types. Finally, it is recommended to accept the article for publication, but the author is requested to carry out the final polishing based on the above remaining issues. I believe that after these adjustments, the article will be more consummate.

**Response 1:** In response to the reviewers' valuable comments, we have substantially revised Sections 4.1 and 4.2 by incorporating detailed descriptions of the spatial distribution patterns and regional heterogeneity of both land use and carbon storage. We are deeply grateful for the reviewers' insightful suggestions, which have considerably improved the scientific rigor and overall quality of our manuscript.

Thanks again for the expert's contribution to this manuscript.
